# Current status and future directions of Lévy walk research

**DOI:** 10.1242/bio.030106

**Published:** 2018-01-15

**Authors:** Andy M. Reynolds

**Affiliations:** Rothamsted Research, Harpenden, Hertfordshire, AL5 2JQ, UK

**Keywords:** Animal movement patterns, Lévy walks, Optimal foraging

## Abstract

Lévy walks are a mathematical construction useful for describing random patterns of movement with bizarre fractal properties that seem to have no place in biology. Nonetheless, movement patterns resembling Lévy walks have been observed at scales ranging from the microscopic to the ecological. They have been seen in the molecular machinery operating within cells during intracellular trafficking, in the movement patterns of T cells within the brain, in DNA, in some molluscs, insects, fish, birds and mammals, in the airborne flights of spores and seeds, and in the collective movements of some animal groups. Lévy walks are also evident in trace fossils (ichnofossils) – the preserved form of tracks made by organisms that occupied ancient sea beds about 252-66 million years ago. And they are utilised by algae that originated around two billion years ago, and still exist today. In September of 2017, leading researchers from across the life sciences, along with mathematicians and physicists, got together at a Company of Biologists' Workshop to discuss the origins and biological significance of these movement patterns. In this Review the essence of the technical and sometimes heated discussions is distilled and made accessible for all. In just a few pages, the reader is taken from a gentle introduction to the frontiers of a very active field of scientific enquiry. What emerges is a fascinating story of a truly inter-disciplinary scientific endeavour that is seeking to better understand movement patterns occurring across all biological scales.

## Abstract (precis)

**Summary:** Movement patterns resembling Lévy walks are found in a wide variety of organisms, from cells to humans. In this Review the latest research into their origins and biological significance is discussed.

*Make things simple, but not too simple*.

  Albert Einstein

*All strange away*.

  Samuel Beckett

Movement is essential to life. It occurs for a myriad of reasons and in a myriad of different ways across a vast range of spatio-temporal scales from the sub-cellular to the whole organism, and from the fleeting to the creeping. Every living thing moves, but each moves in its own idiosyncratic way; ecologists, botanists, physiologists, and cell biologists make strenuous efforts to painstakingly characterise these nuanced, intricate, individual- and context-specific movements. It would seem that marine biologists interested in the predatory movements of sharks would have little to say to immunologists interested in T cells or to ecologists interested in roe deer, mussels and grasses on sand dunes. However, in September of 2017, marine biologists, immunologists and ecologists did gather at a Company of Biologists' Workshop, drawn together because they were seeing seemingly similar patterns of movement. Despite the huge differences in scale, T cells were hunting like aquatic marine predators, which in turn had diving patterns that resembled the dispersal patterns of seeds. Such commonality had not escaped the attention of physicists and mathematicians who, in contrast to colleagues in the life sciences, strive to seek out general principles that apply broadly, despite differences in the underlying details. They too had congregated in an idyllic country house in the English countryside for the Company of Biologists’ Workshop, and this eclectic group were passionately discussing patterns of movement that resemble ‘Lévy walks’.

Lévy walks, named after the French mathematician Paul Lévy, arose in a purely mathematical context in the first half of the last century (Lévy, 1937). They are specialised forms of random walks composed of clusters of multiple short steps with longer steps between them (Fig. 1). This pattern is repeated across all scales with resulting clusters creating fractal patterns that have no characteristic scale. Schlesinger and Klafter (1986) were amongst the first to propose that Lévy walks might be observed in animal movement patterns. They recognised that the fractal properties of Lévy walks can be advantageous during searching, because they reduce the needless revisiting of previously traversed terrain and as a consequence may be under positive selection pressure. This was a revolutionary idea, as it suggested that Lévy walks might be seen across taxa, and represent an innate, evolved, optimal searching strategy. The idea gained traction following the seminal paper of Viswanathan et al. (1996), whose report on the flight patterns of the wandering albatross, *Diomedea exulans*, was the first test of the fledgling ‘Lévy flight foraging hypothesis’ using animals in their natural environments. Later, Viswanathan et al. (1999) demonstrated mathematically that foragers with Lévy walk movement patterns can, indeed, outperform foragers with other kinds of movement patterns. They are particularly advantageous when searching in uncertain or dynamic environments where the spatial scales of searching patterns cannot be tuned to target distributions. The papers of Viswanathan et al. (1996, 1999) led to an explosion of interest in Lévy walks as models of movement pattern data, and shaped much of the subsequent research. However, it subsequently became apparent that many of these early studies, including the seminal study of Viswanathan et al. (1996), had wrongly attributed Lévy walks to some species because inappropriate statistical techniques had been used and movement pattern data had been misinterpreted (Edwards et al., 2007). These issues have now been resolved and in the intervening years there has been an accumulation of evidence that a wide variety of organisms do, after all, have movement patterns resembling Lévy walks. Such movement patterns have been seen, to some extent, in protein motors, trace fossils (the preserved movement patterns of extinct creatures that once lived in ancient sea beds), bacteria, T cells, molluscs, honeybees, a diverse range of aquatic marine predators, and even in human hunter-gatherers (Ariel et al., 2015; Chen et al., 2015; de Jager et al., 2011; Harris et al., 2012; Hays et al., 2012; Korobkova et al., 2004; Raichlen et al., 2014; Reynolds et al., 2007, 2016a,b; Sims et al., 2008, 2014). And now it seems that some wandering albatrosses and other pelagic birds do, after all, fly in a way that Paul Lévy would have appreciated (Humphries et al., 2013; Reynolds et al., 2015; Miramontes et al., 2012).

**Fig. 1.**
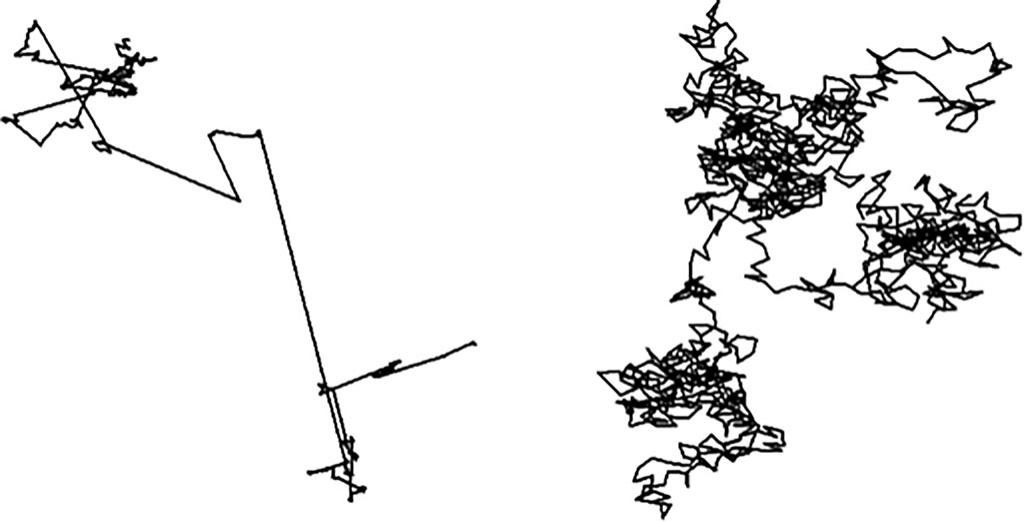
**An example of a Lévy walk (left) and a Brownian walk (right).** The Lévy walk is seen to comprise clusters of multiple short steps with longer steps between them. The longest step is seen to make the dominant contribution to the overall length of the movement pattern. The Brownian walk is seen to comprise many similar steps.

To test for the presence of Lévy walks biologists and ecologists first partition their telemetry data into sequences of ‘steps’ (bouts of near-unidirectional travel) and ‘turns’ or ‘stops’ that break directional persistence. If the step lengths are Gaussian distributed then the most commonly occurring steps will make the dominant contribution to the overall movement pattern (Fig. 1), but this is not so for Lévy walks. A defining hallmark of a Lévy walk is step-length distribution with a ‘heavy’ tail that decays more slowly than a Gaussian distribution. In a Lévy walk, the longest step dominates, dwarfing the contributions from other steps in the movement pattern (Fig. 1). Lévy walks have other remarkable mathematical properties that fascinate mathematicians and physicists but which fall under the radar of biologists and ecologists. It is not the Lévy walks per se that matter to biologists and ecologists, but their essential features that can be approximated by other kinds of movement pattern. Indeed, few scientists would claim that any organisms actually do have Lévy walk movement patterns in the strict mathematical sense, but many would say that some organisms, in some circumstances, do have patterns of movements resembling Lévy walks. As a consequence, there has been a rich dialogue between biologists, ecologists, physicists and mathematicians. Nonetheless, the literature on Lévy walks as models of organism movement patterns, which has mushroomed in the past 20 years, is fractured. Misunderstanding and confusion was almost inevitable given that the subject matter cuts across so many disciplines. The Workshop was convened to resolve these differences and to drive the field of enquiry forward.

For many ecologists and biologists, Lévy walks are a useful concept when confronted with intrinsic multi-scale patterns of movement that are distinctly different from the more prevalent scale-specific patterns of movements, as they provide simple, low parameter descriptions of some movement pattern data. Their association with the presence of an evolutionary attractor (Schlesinger and Klafter, 1986; Viswanathan et al., 1996, 1999) is useful because it leads to new hypotheses and to new methods of analysis, and it has predictive power that ecologists and biologists can work with. Perhaps more importantly has been the push back and resistance to the Lévy flight foraging hypothesis, which has led to the realisation that Lévy walks can arise in situations not related to optimal foraging (Reynolds, 2015a). This strand of Lévy walk research has provided a vivid illustration that the key to understanding the biological, ecological and evolutionary consequences of any movement pattern lies in elucidation of the underlying mechanisms (Levin, 1992; Nathan et al., 2008). The significance of a particular Lévy walk movement pattern can, in fact, vary markedly even across closely related species, and perhaps even within the same organisms under different scenarios (Reynolds, 2015a). Indeed, since their entry into the biological literature (Schlesinger and Klafter, 1986; Viswanathan et al., 1996, 1999), there have been profound changes in our understanding of both the contexts in which Lévy walks can occur and the reasons for their occurrence in these contexts. The Lévy flight patterns of the wandering albatross may, for example, be an inconsequential by-product of odour-cued navigation (Reynolds et al., 2015). Seeds are another striking example. Some seed dispersal patterns will bear the hallmarks of a Lévy walk only after becoming airborne under particular weather conditions at take-off (Reynolds, 2013a). Intriguingly, these movement patterns maximise the likelihood of dispersing to the nearest unoccupied site, thereby maximising expected fitness on landing (Reynolds, 2013a). Other recent studies suggest that the alga *Chamydomonas reinhardtii* is perhaps the most ancient example of an organism benefiting from Lévy movements when foraging; these single eukaryotic cells originated around two billion years ago and still exist today. *C. reinhardtii* swim with two flagella, and when in a dilute suspension, the flagella induce flows that cause nutrient particles to acquire Lévy signatures and thereby undergo enhanced diffusion (Leptos et al., 2009). This is biologically significant because the movement does not significantly increase the likelihood of the alga encountering nutrients; these microorganisms live at low Reynolds numbers (a dimensionless number used to predict flow patters in different situations) and so carry most of their local environment with them, which only gradually falls behind (Purcell, 1977). Transport of nutrients towards the alga is entirely controlled by diffusion (mixing). The enhancement of diffusion due to nutrients moving on Lévy walks caused by the movement of flagella may be purely coincidental, but it does illustrate that the biological significance of Lévy walks is ancient and not confined to the kind of foraging processes originally envisaged by Schlesinger and Klafter (1986) or Viswanathan and co-workers ( 1996, 1999). It seems that advantageous searching is the tip of an explanatory iceberg, as Lévy walks can and do arise in a variety of search behaviours unrelated to foraging, and can even arise in movement behaviours that are not related to searching. It seems that Lévy walks as models of movement patterns are too rich of a concept to be constrained by an optimal foraging hypothesis or indeed by any simple principle; going forward we should be much more ambitious about the scope of Lévy walks and their impact on ecological and biological processes.

The emerging picture is a pluralistic one (Reynolds, 2015a) (Table 1). This will come as no surprise to ecologists who strive to describe, as best as possible, animal behaviours without the means for general conclusions. Lévy walk movement patterns may be common and follow general rules that apply widely and are context independent, but the underlying generative behaviours (processes) are context specific. The plethora of generative behaviours falls squarely within the domain of the ecologists and biologists who regularly face such complexity. The patterns, however, can be made simple (but not too simple) and so can be tackled by physicists and mathematicians with their arsenal of analytical techniques, as can the linkages between behaviours and patterns. It is because of this synergy of ideas that Lévy walk research remains so vibrant and exciting.

**Table 1.**
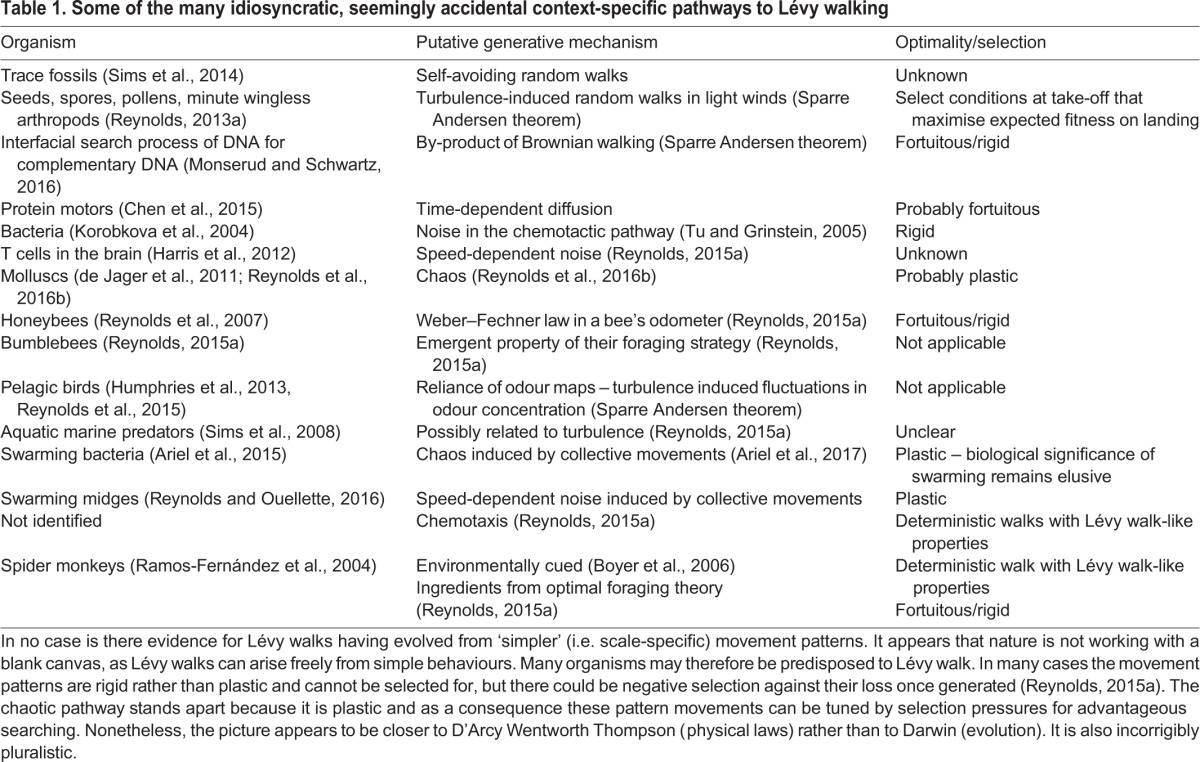
**Some of the many idiosyncratic, seemingly accidental context-specific pathways to Lévy walking**

Nature repeatedly reveals the limits of our imagination. Lévy walks once thought to be the preserve of probabilistic foragers have now been identified in the movement patterns of human hunter-gatherers (Raichlen et al., 2014). This seems remarkable given that ‘humans are the most cognitively complex foragers on Earth’ (Raichlen et al., 2014). The defining characteristics of Lévy walks, heavy-tailed distributions, have also broken free from their spatial confines and have been found to characterise the durations of pauses between movement bouts. The movements of many organisms are interspersed with pauses or rests. Such intrinsic spontaneous intermittency is, for example, evident in the activity patterns of some sympatric predator species (including cephalopods, elasmobranch and teleost fish) under natural and controlled conditions (Wearmouth et al., 2014). The pause durations in these diverse predatory groups are, in fact, well approximated by heavy-tailed power-law distributions over data ranging from seconds to hours. It is possible that these temporal (waiting time) scaling laws are the parallel in ambush predators of the spatially scale-invariant Lévy walk patterns that seem common among animals that move continually during searching. This would suggest that power-law scaling applies generally across taxa with divergent foraging strategies, ranging from highly mobile pursuit predators (i.e. Lévy walkers) to the less mobile ambush predators. As with Lévy walk movement patterns, the emerging picture in the temporal domain is a pluralistic one. Heavy-tailed waiting times have been seen in T cells (Harris et al., 2012) and in shearwaters (Reynolds et al., 2016a). More recently, Lévy walk movement patterns have been found to emerge from collective behaviours, and are evident to some extent in swarming bacteria (Ariel et al., 2015), in midge swarms (Reynolds and Ouellette, 2016), in termite broods (Miramontes et al., 2014), and in fish (Murakami et al., 2015).

The key question is now is not whether some organisms have Lévy walk movement patterns, but when and why they do. The challenge is to put Lévy walk movement patterns into context by formulating appropriate hypotheses. Some of the most significant advances have come when biologists, physicists and mathematicians have worked together to overcome differences in focus, approach and language; but some of those coming from the life sciences, especially from ecology, struggle with the very notion of Lévy walks as generic emergent patterns of movement that can come from the complex ecosystems they so meticulously describe. They instead prefer more conventional models founded on scale-specific random walks (Brownian walks), and multiphasic walks that describe animals switching between different types of movements as they engage in different activities or enter changes in the landscape. This is also true in cell biology, where models of motility are often inspired by their fit to the experiment. Many of the discussions at the Workshop (sometimes heated, but always sincere) were focused on this apparent schism. In many cases, it seems that the dichotomy is a false one, and all that is required are conceptual leaps of the imagination. In many regards, Lévy walks are no stranger than Brownian motion, multiphasic walks, or the models of cell motility.

In 1828 the Scottish botanist Robert Brown reported that minuscule pollen particles suspended in still water have seemingly random movements. Einstein's (1905) subsequent mathematical description of these random ‘Brownian’ movement patterns has been hugely successful and now lies at the heart of the ‘correlated random walk paradigm’ – the dominant conceptual framework for modelling animal movement patterns (Turchin, 1998). But the humble pollen has other tales to tell, which show that Lévy movements are pertinent even in the simplest of situations. Occasionally, one of Robert Brown's pollen grains would come into contact with the bottom of his dish. Standard Brownian walk theory predicts that these landing points will form Lévy patterns (Reynolds, 2011). It seems that Robert Brown and Einstein both came very close to discovering the patterns of movement that we now call ‘Lévy walks’. Lévy walks also abound once the pollen grains are liberated from watery confines and are at the mercy of the wind (Reynolds, 2011). This is also true of seeds, spores and small wingless arthropods. Although these airborne movements are clearly divorced from searching, they are not without consequence as they result in patchy, fractal-like, spatial population structures very different from the structure of a homogeneous front produced by Brownian movements (Reynolds, 2011). Analogous behaviour has recently been observed in single-molecule tracking experiments of proteins, polymer and small molecules (Skaug et al., 2013), as well as in the interfacial search process of DNA for complementary DNA (Monserud and Schwartz, 2016). The Brownian-like flight patterns of bumblebees (Lenz et al., 2012) might also lead to Lévy-like landing (exploration) patterns. In each of these settings, Brownian walks and Lévy walks are seen to go hand-in-hand and characterise different facets of the movement patterns, albeit seen from different perspectives. The Langevin equation, the much-studied bedrock of Brownian walk theory, does, in fact, describe a Lévy walk (albeit with truncation) (Reynolds, 2010a). Recent studies also suggest that some animals (e.g. Australian desert ants and a variety of molluscs) (de Jager et al., 2012; Reynolds et al., 2016b; Reynolds, 2014) approximate Lévy walks as multiphasic walks. This in turn suggests that Lévy walks and multiphase walks are not necessarily competing models of movement patterns as suggested by Benhamou (Benhamou, 2007), but rather are different ways of approximating an optimal foraging behaviour. In this regard, Lévy walks can be seen as a simple, approximate, integrative description of animal movement. Multiphasic walks, on the other hand, potentially provide more complex mechanistic models, pointing at a possible way by which animals could approximate scale-free movement patterns (Reynolds, 2013c).

In each of the above cases, Lévy walks have been hiding in plain sight. Cell motility is another example of this. The large quantities of data from computer-aided cell tracking experiments can and have been used to accurately parameterise now-standard models for spontaneous cell movements (Selmeczi et al., 2005, 2008). The very close agreement between these models and experimental data does not seem to add much to our understanding of these cells beyond demonstrating that their motilities can be modelled phenomenologically, but in the physics literature such models are known to produce Lévy walk movement patterns (Reynolds, 2010b).

Lévy walks entered the biological literature more than 20 years ago. We now have a much better understanding of the very diverse but context-specific conditions under which Lévy walks can and do arise, many of which are unrelated to advantageous foraging. This historical arc mirrors, to some extent, that of ‘self-organized criticality (SOC)’ in biology – a concept first developed in physics and mathematics and championed by Per Bak (Bak et al., 1987), and then conjectured to exist in biology. There are now plenty of good biological examples of SOC, but the promised generality has not been seen. Going forward, we must continually be open to reinterpretations and new possibilities, as exemplified, for example, by Namboodiri et al. (2016), who showed how Lévy walk searching might arise in cognitive searchers that learn from their environment. But to make progress, theoretical developments must be anchored in biology and be underpinned mechanistically by process- and behavioural-based models that account for environmental feedback. Analyses must be put into context, and it is likely that much can be gained by going beyond characterizations of movement patterns solely in terms of step-length distributions, as exemplified recently by an analysis of human mobility patterns (Gallotti et al., 2016).

During the Workshop, we learnt of some of these new developments. We learnt that Lévy walks may be implicated in sand-dune formation; that some waterborne seeds have Lévy dispersal characteristics by virtue of their size distributions; that the rich variety of Lévy walk movements seen in roe deer in their natural environments are mirrored by those seen in the laboratory in genetically modified *Drosophila* larvae that cannot sense their environment; that Lévy walks can emerge from optimal searching strategies rather than being an optimal searching strategy per se; that T cells deform markedly during movement suggesting that fruitful connections can be made with the physics literature on the emergence of Lévy walks in highly deformable active, self-propelled particles (Matsuo et al., 2013), a connection that may help explain why T cells perform Lévy walks in the brain (Harris et al., 2012) but not in other tissues (Banigan et al., 2015, Fricke et al., 2016); and that social interactions can give rise to Lévy walks in animal groups. We also learnt of the wider literature on anomalous diffusion, of which Lévy walks are just one part. These and other advances will appear in print in due course. In the meantime, the interested reader can find out more in a recent technical review (Reynolds, 2015a) and in the associated commentaries (Bartumeus, 2015; Boyer, 2015; Cheng, 2015; da Luz et al., 2015; Focardi, 2015; Humphries, 2015; MacIntosh, 2015; Miramontes, 2015; Sims, 2015; Reynolds, 2015b), which for the most part were written by the delegates of the Workshop. For researchers, open questions abound. If Lévy walks are a biologically accessible and generally advantageous searching strategy for probabilistic foragers, then where are all the Lévy walkers? Many insect herbivores must literally bump into a host plant before they recognise it is food (Turchin, 1998) and yet evidence for Lévy walks in insects is limited. Aquatic marine predators have provided some of the most striking evidence for Lévy walks (Sims et al., 2008) and yet the underlying generative mechanism remains elusive. This search for the generative mechanism may have been thwarted by the assumption that the one-dimensional Lévy diving patterns are indicative of three-dimensional Lévy movement patterns. This is an over-interpretation of the current observations. The possibility that the Lévy walks made by mud snails and other molluscs have their mechanistic origins in chaotic neuronal processes calls (Reynolds et al., 2016b) for new research on the coupling between neurobiology and motor properties. Analysis of individuals has often been set aside in favour of using population-level analyses, making any discussion of selection, which acts on individuals, problematic (MacIntosh, 2015). This is changing (Humphries et al., 2013, 2016; Reynolds et al., 2015), but more needs to be done to access empirically intra- and interspecific variation. And so the list of questions and challenges goes on.

It would take the proverbial monkey randomly typing at a keyboard aeons to produce this report; before its emergence there would be seemingly endless pages of gibberish – strings of random symbols would be punctuated by the occasional spaces. But despite appearances there would be structure in this madness. The lengths of the strings will follow a heavy-tailed power-law distribution that is the defining feature of a Lévy walk (Miller, 1957) of the kind found to optimise random search processes (Viswanathan et al., 1999). This would remain the case even if the keys were struck with unequal probabilities (Perline, 1996). These Lévy walks, like the many described herein, arise freely (and perhaps surprisingly) from the simplest of processes, have been hiding in plain sight, are rigid, and accidentally optimal. It should now come as no surprise that some organisms will sometimes have movement patterns that resemble Lévy walks. Lévy walks are not some exotic form of movement pattern divorced from reality, but one that is entirely natural. The astute reader may have even discerned the presence of Lévy-like patterns lurking in my prose (Zipf, 1935).
